# Carbon Isotope Composition of Nighttime Leaf-Respired CO_2_ in the Agricultural-Pastoral Zone of the Songnen Plain, Northeast China

**DOI:** 10.1371/journal.pone.0137575

**Published:** 2015-09-10

**Authors:** Haiying Cui, Yunbo Wang, Qi Jiang, Shiping Chen, Jian-Ying Ma, Wei Sun

**Affiliations:** 1 Key Laboratory of Vegetation Ecology, Ministry of Education, Institute of Grassland Science, Northeast Normal University, Changchun, Jilin Province, P. R. China, 130024; 2 State Key Laboratory of Vegetation and Environmental Change, Institute of Botany, Chinese Academy of Sciences, Beijing, P. R. China, 100093; 3 Xinjiang Institute of Ecology and Geography, Chinese Academy of Sciences, Urumqi, P. R. China, 830011; Universidade Federal de Viçosa, BRAZIL

## Abstract

Variations in the carbon isotope signature of leaf dark-respired CO_2_ (δ^13^C_R_) within a single night is a widely observed phenomenon. However, it is unclear whether there are plant functional type differences with regard to the amplitude of the nighttime variation in δ^13^C_R._ These differences, if present, would be important for interpreting the short-term variations in the stable carbon signature of ecosystem respiration and the partitioning of carbon fluxes. To assess the plant functional type differences relating to the magnitude of the nighttime variation in δ^13^C_R_ and the respiratory apparent fractionation, we measured the δ^13^C_R_, the leaf gas exchange, and the δ^13^C of the respiratory substrates of 22 species present in the agricultural-pastoral zone of the Songnen Plain, northeast China. The species studied were grouped into C_3_ and C_4_ plants, trees, grasses, and herbs. A significant nocturnal shift in δ^13^C_R_ was detected in 20 of the studied species, with the magnitude of the shift ranging from 1‰ to 5.8‰. The magnitude of the nighttime variation in δ^13^C_R_ was strongly correlated with the daytime cumulative carbon assimilation, which suggests that variation in δ^13^C_R_ were influenced, to some extent, by changes in the contribution of malate decarboxylation to total respiratory CO_2_ flux. There were no differences in the magnitude of the nighttime variation in δ^13^C_R_ between the C_3_ and C_4_ plants, as well as among the woody plants, herbs and graminoids. Leaf respired CO_2_ was enriched in ^13^C compared to biomass, soluble carbohydrates and lipids; however the magnitude of enrichment differed between 8 pm and 4 am, which were mainly caused by the changes in δ^13^C_R_. We also detected the plant functional type differences in respiratory apparent fractionation relative to biomass at 4 am, which suggests that caution should be exercised when using the δ^13^C of bulk leaf material as a proxy for the δ^13^C of leaf-respired CO_2_.

## Introduction

The stable C isotope composition (δ^13^C) has been widely used to trace the carbon flow within ecosystem components or between the ecosystem and the atmosphere [1–7]. The applications of stable carbon isotope technique require a mechanistic understanding of the temporal and spatial variation in the δ^13^C signature of component fluxes [8–10]. As an important component of ecosystem carbon fluxes, leaf respired CO_2_ has been reported to vary substantially in its carbon isotope composition at diurnal timescale [11–15]. Unfortunately, we still lack a comprehensive understanding of the processes controlling the dynamics in the δ^13^C signature of leaf respired CO_2_ [9, 16].

It has been extensively reported that δ^13^C_R_ varied substantially, up to 14.8‰, on a diurnal timescale [12, 13, 17, 18]. Several mechanisms have been developed to explain short-term variation in δ^13^C_R_. Firstly, intramolecular ^13^C distribution is not homogeneous in hexose molecules, which combined with changes in the relative contribution of the metabolic pathways to the respiration could lead to variation in δ^13^C_R_ [14, 19–22]. Secondly, shifts in δ^13^C_R_ can be attributed to the changes in the contribution of malate (^13^C-enriched) decarboxylation to the overall respiratory flux [8, 23]. Thirdly, changes in the use of the respiratory substrates having different δ^13^C may subsequently affect δ^13^C_R_ [16, 18]. Finally, short variation in carbohydrate pool size may also influence δ^13^C_R_ through affecting the allocation of respiratory intermediates [24]. The results of previous study showed substantial intraspecific and interspecific differences in the amplitude of the short-term variation in δ^13^C_R_ [14, 18, 25]. Intraspecific differences in the range of the diurnal variation in δ^13^C_l_ are caused mainly by the availability of resources associated changes in the substrate availability and the allocation of the respiratory intermediates [25]. The findings of previous studies by Werner et al. (2007) and Priault et al. (2009) indicated large diurnal variations in the δ^13^C_R_ of slow-growing aromatic plants, whereas no apparent diurnal shift was found in the δ^13^C_R_ in temperate trees and fast-growing herbs. These results highlight the potential plant functional type differences relating to the extent of the variation in δ^13^C_R_ on a diurnal timescale [11, 14, 22]. However, plant functional type differences in the magnitude of short-term variation in δ^13^C_R_ need to be further explored, especially for C_3_ and C_4_ species which differed substantially in the magnitude of heterogeneous ^13^C distribution within hexose molecules [21, 26].

Leaf dark-respired CO_2_ is often enriched in ^13^C compared with leaf bulk tissue or other potential respiratory substrates, such as starch, soluble carbohydrates, and others [27–29]. This phenomenon is attributed mainly to non-homogeneous ^13^C distribution among the carbon atoms within the hexose molecules and the incomplete oxidation of hexoses. However, the phenomenon is also attributed partially to the utilization of isotopically different respiratory substrates [21, 24, 30]. Because of the intramolecular ^13^C/^12^C differences, CO_2_ that evolved from pyruvate decarboxylation contains more ^13^C relative to that derived from acetyl-CoA oxidation [31, 32]. Depending on substrate availability, ^13^C-depleted acetyl-CoA could be used for the biosynthesis of lipids and secondary compounds, or decarboxylation in the TCA cycle to generate adenosine triphosphate (ATP). Therefore, the incomplete oxidation of acetyl-CoA could lead to ^13^C enrichment in leaf dark-respired CO_2_ relative to respiratory substrates. The plant functional types differed substantially with regard to the allocation of ^13^C-depleted compounds. Trees, for instance, allocated proportionally more acetyl-CoA to the synthesis of lipids and lignin than did grasses [14, 26]. This could lead to the leaf-respired CO_2_ in trees being more enriched in ^13^C in comparison with the putative respiratory substrates or the bulk leaf material. Indeed, plant functional type differences in respiratory apparent fractionation have been reported between C_3_ and C_4_ species, but not between woody plants and C_3_ herbs [15, 16]. However, these findings were derived from meta-analysis of results obtained under various growing habitats and using different methods, which makes the conclusions unreliable. Therefore, the existence of plant functional type differences with regard to respiratory apparent fractionation needs to be further verified and, if they do exist, we need to incorporate these into the flux partitioning.

The vegetation of the agricultural-pastoral zone of the Songnen Plain is characterized as mosaics of grassland and cultivated land, dominated mainly by C_3_ species and C_4_ species, respectively. We measured the δ^13^C_R_ and δ^13^C of the putative primary respiratory substrates of 22 species at 8 pm and 4 am. The leaf gas-exchange parameters, including net assimilation rate, stomatal conductance, and nighttime respiration rate were also measured. The plant species were grouped into C_3_ and C_4_ species, trees, graminoids, and herbs. The objectives of the present study were to assess the plant functional type differences in the range of nighttime variation in δ^13^C_R_ and the respiratory apparent fractionation relative to the bulk leaf material and the putative respiratory substrates.

## Materials and Methods

### Ethics Statement

No specific permissions were required for the field studies described, because the Songnen Grassland Ecological Research Station is a department of the Northeast Normal University. No specific permissions were required for the study either, as it was conducted in accordance with the guidelines set by the Northeast Normal University. No specific permissions were required for the locations or the activities. No location was privately owned or protected in any way, and the field studies did not involve endangered or protected species.

### Study site

**Fig 1:**
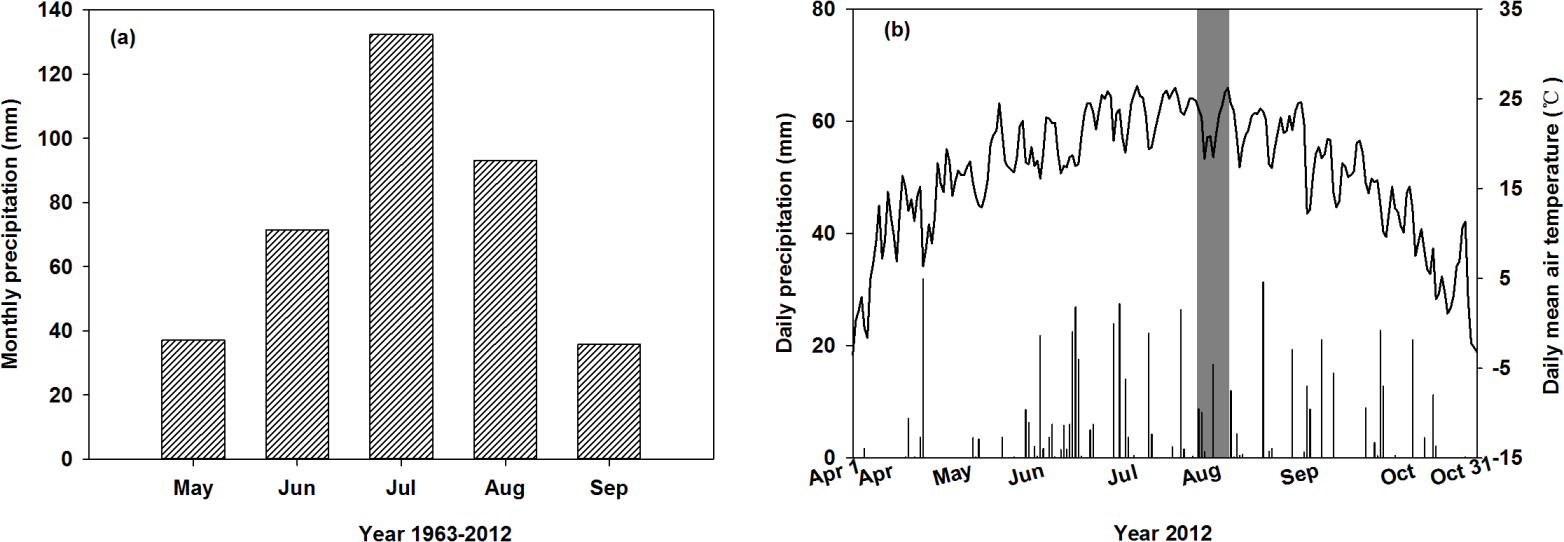
Growing season average monthly precipitation (mm) from 1963 to 2012 (a), and daily precipitation (mm) and daily mean air temperature (°C) from April 1^th^ to October 31^th^ in 2012 (b). The shaded area in panel b denotes the field sampling period.

The study was conducted in the agricultural-pastoral zone of the Songnen Plain (44°40′–44°44′N, 123°44′–123°47′E, elevation 138–144 m) in northeast China. The study area has a semiarid continental climate, with a mean annual temperature of 6.4°C. The mean annual rainfall is 471 mm, with over 70% of the precipitation occurs from June to August (Fig 1A). Drought, especially spring drought, occurs frequently in the studied area during the growing season; however there is no fixed drought period. The duration of the frost-free season is approximately 150 days. Detailed information on daily precipitation and daily temperature in 2012 is provided in Fig 1B. The main soil type of the study area is chernozem, with a soil organic carbon content of 2.0% and a soil total nitrogen content of 0.15% [33]. The vegetation in the study area is characterized as mosaics of grassland and cultivated land. The grassland is dominated by *Leymus chinensis*, while *Phragmites australis* and *Chloris virgata* are present in abundance[34]. The major crops on the cultivated land are *Zea mays*, *Setaria italica*, *Helianthus annuus*, and *Sorghum bicolor*. We studied 22 plant species, representative of the major species present on the grassland and the cultivated land. The details of the species studied are provided in Table 1. The field studies were conducted from July 25^th^ to August 5^th^ in 2012, which is the peak biomass season for the study area. None of the studied species showed signs of senescence. Information on the development stage of the studied species is provided in Table 1.

**Table 1:** Information on species studied. List of plant species studied, and their photosynthetic pathway, classification and phenological phase.

Species	Photosynthetic pathway	Functional type	Class	Phenological phase
*Leymus chinensis*	C_3_	Graminoids	Monocotyledoneae	Fructescence
*Phragmites australis*	C_3_	Graminoids	Monocotyledoneae	Florescence
*Lespedeza bicolor*	C_3_	Trees	Dicotyledoneae	Florescence
*Malus asiatica*	C_3_	Trees	Dicotyledoneae	Fructescence
*Populus simonii*	C_3_	Trees	Dicotyledoneae	Post-fruiting vegetative stage
*Prunus salicina*	C_3_	Trees	Dicotyledoneae	Fructescence
*Chenopodium glaucum*	C_3_	Herbs	Dicotyledoneae	Florescence
*Glycine max*	C_3_	Herbs	Dicotyledoneae	Flowering and pod formation stage
*Helianthus annuus*	C_3_	Herbs	Dicotyledoneae	Florescence
*Saussurea amara*	C_3_	Herbs	Dicotyledoneae	Florescence
*Vigna radiata*	C_3_	Herbs	Dicotyledoneae	Flowering and pod formation stage
*Vigna unguiculata*	C_3_	Herbs	Dicotyledoneae	Flowering and pod formation stage
*Xanthium sibiricum*	C_3_	Herbs	Dicotyledoneae	Florescence
*Chloris virgata*	C_4_	Graminoids	Monocotyledoneae	Florescence
*Echinochloa crusgalli*	C_4_	Graminoids	Monocotyledoneae	Florescence
*Hemarthria altissima*	C_4_	Graminoids	Monocotyledoneae	Heading stage
*Panicum miliaceum*	C_4_	Graminoids	Monocotyledoneae	Florescence
*Setaria italica*	C_4_	Graminoids	Monocotyledoneae	Florescence
*Setaria viridis*	C_4_	Graminoids	Monocotyledoneae	Florescence
*Sorghum bicolor*	C_4_	Graminoids	Monocotyledoneae	Elongation stage
*Zea mays*	C_4_	Graminoids	Monocotyledoneae	Tasseling stage
*Amaranthus retroflexus*	C_4_	Herbs	Dicotyledoneae	Florescence

### Leaf gas-exchange measurement

Leaf photosynthesis was measured every three hours, from 6 am to 6 pm, using a LI-6400 infrared gas-exchange analyzer (LI-COR Biosciences Inc., Lincoln, NE, USA). We selected fully expanded leaves from the sunny side for leaf photosynthetic rate measurements. Before each measurement, the leaf chamber conditions were set to match the environmental conditions, including the photosynthetically active radiation, air temperature, and relative humidity. Leaf respiration rates at 8 pm and 4 am were measured under zero light intensity. The leaf photosynthesis and respiration measurements for each species were conducted on five randomly selected plants.

### Collection of leaf-respired CO_2_

Leaf-respired CO_2_ was collected in the field using the gas-tight syringe incubation method [11]. In brief, young and fully expanded leaves (comparable to those used to measure the photosynthesis and the respiration rates), were detached and placed inside a gas-tight syringe barrel. The syringe barrel was subsequently flushed with CO_2_-free air to remove the background CO_2_. The syringe barrel was then sealed and incubated for 15 min to allow for the buildup of leaf-respired CO_2_. After the incubation, 5 ml of air, containing leaf-respired CO_2_, was injected into a 12 ml vial. The vial was filled with helium and fitted with septum caps. For each studied species, the leaf-respired CO_2_ was collected twice (8 pm and 4 am) during a single night. For the period of field sampling, sunset time ranged from 7:17 pm to 7:04 pm, therefore the collection of leaf-respired CO_2_ at 8 pm was conducted at least 40 min after sunset and in the darkness. The leaf-respired CO_2_ was sampled each time on five randomly selected plants or populations.

### Lipids, soluble carbohydrates, and starch extractions

Simultaneously with the leaf-respired CO_2_ sampling, we collected leaves for measuring the carbon isotope composition of leaf bulk materials and potential respiratory substrates. The collected leaves were comparable (age and canopy position, etc.) with those used for the leaf photosynthesis measurements. The collected leaves were immersed in liquid nitrogen to stop the metabolic activities. They were subsequently stored at -80°C in a deep freezer before being freeze-dried with a Labconco freeze drier (Labconco Kansas City, MO, USA). A ball mill (MM 400 Retsch, Haan, Germany) was used to grind the freeze-dried leaves into fine powder.

We used the protocols described by Wanek et al. (2001) and Göttlicher et al. (2006) [35, 36] for the extraction of lipids, soluble carbohydrates, and starch. In brief, the powdered leaf material was extracted with methanol/chloroform/water (MCW; 12:5:3, v/v/v). After centrifugation, the chloroform and the deionized water were added to the supernatant for phase separation. The chloroform phase (containing lipids) was dried in a ventilation device and subsequently analyzed for the carbon isotope composition.

The upper water phase (containing soluble carbohydrates) was transferred into a reaction vial and oven dried for carbohydrate extraction. The residue was re-dissolved in deionized water. After centrifugation, the phase containing the soluble carbohydrates was purified by an ion-exchange column, including both anion- and cation-exchange resin. The eluent was collected and oven dried, and the carbon isotope composition was subsequently analyzed, using an isotope ratio mass spectrometer.

The plant materials were re-extracted with MCW to remove the residual lipids and the soluble carbohydrates. After the starch in the plant materials had been gelatinized and hydrolyzed, the aqueous phase was separated from the pellet by centrifugation. The upper aqueous phase was subsequently purified by a centrifugal filter unit (Microcon YM-10; Millipore, Billerica, MA, USA). The filtrate containing glucose originated from starch was oven dried in a tin capsule and then analyzed for the carbon isotope composition.

### Carbon isotope ratio analysis

All carbon isotope ratio analyses were performed using an isotope ratio mass spectrometer (Isoprime 100, Isoprime Ltd., Manchester, UK). The precision of repeated δ^13^C measurements on solid and gaseous working standards was < 0.1‰. The C isotope ratios are reported in parts per thousand relative to Vienna Pee Dee Belemnite (VPDB) as

$$\delta^{13}C\left(‰\right)=\;（R_{\text{sample}}/R_{\text{standard}}-1）\;\times 1,000$$

### Respiratory apparent ^13^C/^12^C fractionation

Respiratory apparent ^13^C/^12^C fractionation, relative to potential respiratory substrates, was calculated as:

$$\Delta_{R,X}=\frac{\delta^{13}{\text{C}}_{X}-\delta^{13}{\text{C}}_{R}}{1+\delta^{13}{\text{C}}_{R}}$$ (1)

where Δ_R,X_ represents the respiratory apparent ^13^C/^12^C fractionation, relative to substrate X, and δ^13^C_X_ represents the carbon isotope composition of substrate X. X represents the leaf bulk materials, lipids, starch, or soluble carbohydrates, while δ^13^C_R_ represents δ^13^C of the leaf-respired CO_2_.

### Statistical analysis

We used one-way analysis of variance (ANOVA) to assess the differences between the photosynthetic pathways in the range of the nighttime variation of δ^13^C_R_, the leaf net CO_2_ assimilation rate (*A*), stomatal conductance (*g*_s_), *C*_i_/*C*_a_, and the respiratory rate (*R*). Linear regression analysis was employed to assess the dependence of the magnitude of the nighttime variation in δ^13^C_R_ on the cumulative carbon assimilation. One-way analysis of variance was conducted to assess the plant functional type differences with regard to the leaf gas-exchange parameters, the magnitude of the nocturnal shifts in δ^13^C_R_, and the nighttime respiratory apparent fractionation. The Statistical Package for the Social Science (SPSS) (version 13.0, IBM, Armonk, NY, USA) was used for all statistical analyses. Data are reported as mean ± 1 standard error.

## Results

### Leaf gas exchange

For the species studied, the maximum leaf net CO_2_ assimilation rate (*A*max) in the C_3_ species varied from 15.1±1.2 to 38.4±5.8 μmol m^-2^ s^-1^; whereas it ranged from 22.3±1.7 to 33.7±1.6 μmol m^-2^ s^-1^ in the C_4_ plants (Table 2). The average *A*max value in the C_4_ plants (28.1 μmol m^-2^ s^-1^) was greater than it was in the C_3_ plants (22.4 μmol m^-2^ s^-1^) (Table 3). The mean leaf net CO_2_ assimilation rate (Mean *A*) varied substantially between 9.6±0.4 and 24.0±0.5 μmol m^-2^ s^-1^ (Table 2). There were no statistically significant differences in Mean *A* between the C_3_ and C_4_ species (Table 3). Moreover, no apparent differences in *A*max and Mean *A* were detected among the different plant functional types (Table 3).

**Table 2:** Leaf gas-exchange data. Maximum leaf net CO_2_ assimilation rate **(** *A*max, μmol m^-2^ s^-1^ **)**, mean leaf net CO_2_ assimilation rate **(**Mean *A*, μmol m^-2^ s^-1^ **)**, maximum stomatal conductance (*g*_s_max, mol m^-2^ s^-1^), mean ratio of intercellular air space to ambient CO_2_ concentration (Mean *C*_i_/*C*_a_), and nighttime mean respiratory rate (Mean *R*, μmol m^-2^ s^-1^) of the species studied. Data are reported as mean ± 1 SE.

Species	*A*max	Mean *A*	*g*_s_max	Mean *C*_i_/*C*_a_	Mean *R*
*L*. *chinensis*	19.9±3.99	11.8±0.82	0.295±0.022	0.75±0.01	1.32±0.16
*P*. *australis*	17.2±0.35	11.8±0.35	0.268±0.011	0.71 ±0.01	0.71 ±0.08
*L*. *bicolor*	22.6±1.87	12.2±0.67	0.586±0.047	0.78±0.01	2.07 ±0.26
*M*. *asiatica*	22.3±0.90	17.0±0.63	0.446±0.017	0.69±0.01	0.74 ±0.09
*P*. *simonii*	23.2±0.32	17.1±0.52	0.511±0.041	0.73±0.03	1.22 ±0.11
*P*. *salicina*	15.3±1.65	11.2±0.75	0.531±0.057	0.65±0.01	0.51 ±0.06
*C*. *glaucum*	28.8±1.04	21.1±0.43	0.943±0.051	0.80 ±0.01	1.28 ±0.13
*G*. *max*	17.0±0.54	10.8±0.49	0.098±0.005	0.20±0.03	1.45±0.11
*H*. *annuus*	21.1±0.13	13.1±0.28	0.128±0.002	0.44 ±0.01	1.10 ±0.10
*S*. *amara*	15.1±1.20	9.6 ±0.36	0.443±0.022	0.82 ±0.01	0.73 ±0.17
*V*. *radiata*	26.3±2.98	14.8±1.64	0.247±0.009	0.81 ±0.01	2.22±0.17
*V*. *unguiculata*	23.9±4.90	15.2±2.66	0.712±0.072	0.59 ±0.14	1.54 ±0.17
*X*. *sibiricum*	38.4±5.80	21.2±1.20	0.814±0.083	0.79 ±0.02	2.12 ±0.13
*C*. *virgata*	28.1±1.71	15.5±1.14	0.179±0.005	0.42 ±0.03	0.91 ±0.13
*E*. *crusgalli*	22.6±1.34	13.1±0.20	0.212±0.017	0.49 ±0.03	0.87 ±0.08
*H*. *altissima*	22.3±1.68	13.8±1.57	0.146±0.015	0.49 ±0.03	1.36 ±0.24
*P*. *miliaceum*	27.9±0.31	18.1±0.28	0.204±0.019	0.41 ±0.03	1.54 ±0.07
*S*. *italica*	30.8±2.27	18.0±0.63	0.247±0.013	0.41 ±0.03	0.82 ±0.40
*S*. *viridis*	27.6±1.88	15.3±0.57	0.199±0.007	0.49 ±0.01	1.15 ±0.11
*S*. *bicolor*	26.2±1.34	17.0±0.47	0.183±0.034	0.27 ±0.02	1.45 ±0.16
*Z*. *mays*	33.7±1.62	23.6 ±0.93	0.367±0.051	0.54 ±0.06	0.82 ±0.05
*A*. *retroflexus*	33.3±0.53	24.0±0.49	0.520±0.040	0.59 ±0.03	1.80 ±0.10

**Table 3:** Statistical data. The *df* and *P* values from the photosynthetic pathway and the plant functional type differences in the maximum leaf net CO_2_ assimilation rate **(** *A*max, μmol m^-2^ s^-1^ **)**, the mean leaf net CO_2_ assimilation rate **(**Mean *A*, μmol m^-2^ s^-1^ **)**, maximum stomatal conductance (*g*_s_max, mol m^-2^ s^-1^), mean ratio of leaf internal to ambient CO_2_ concentration (Mean C_i_/C_a_), nighttime mean respiration rate (Mean *R*, μmol m^-2^ s^-1^), magnitude of nocturnal shift in δ^13^C_R_ (Variation in δ^13^C_R_), and respiratory apparent ^13^C/^12^C fractionation (‰) comparative to biomass (Δ_R, biomass_), soluble carbohydrates (Δ_R, sugar_), starch (Δ_R, starch_) and lipids (Δ_R, lipid_) at 8 pm and 4 am, respectively.

	Photosynthetic pathway		Functional type	
	*df*	*P*	*df*	*P*
*A*max	1	**0.03**	2	0.40
Mean *A*	1	0.39	2	0.77
*g*_s_ max	1	**0.02**	2	**0.02**
Mean C_i_/C_a_	1	**<0.01**	2	0.09
Mean R	1	0.59	2	0.14
Variation in δ^13^C_R_	1	0.07	2	0.29
Δ_R, biomass-8pm_	1	0.90	2	0.78
Δ _R, biomass-4am_	1	**0.02**	2	0.52
Δ_R, sugar-8pm_	1	**<0.01**	2	0.09
Δ _R, sugar-4am_	1	0.22	2	0.27
Δ _R, starch-8pm_	1	0.89	2	0.60
Δ _R, starch-4am_	1	0.11	2	0.33
Δ _R, lipid-8pm_	1	**<0.01**	2	**<0.01**
Δ _R, lipid-4am_	1	**<0.01**	2	**<0.01**

The maximum daily stomatal conductance (*g*_s_max) varied from 0.10±0.01 to 0.94±0.05 mol m^-2^ s^-1^. Moreover, the apparent differences in *g*_s_max were detected between the C_3_ and the C_4_ species (Tables 2 & 3). In addition, we detected significant differences in *g*_s_max among the woody plants, forbs, and graminoids (Table 3). The mean respiration rate (Mean *R*) varied from 0.51±0.06 to 2.22±0.17 μmol m^-2^ s^-1^ (Table 2). There was no significant difference in the mean *R* among the functional groups, and it did not differ between the C_3_ and the C_4_ species either (Table 3). The mean ratio of the leaf intercellular air space to the ambient CO_2_ concentration (Mean *C*_i_/*C*_a_) varied from 0.20±0.03 to 0.81±0.01 and 0.27±0.02 to 0.59±0.03 for the C_3_ and C_4_ species, respectively. The mean *C*_i_/*C*_a_ in the C_3_ plants was significantly greater than it was in the C_4_ plants (Tables 2 & 3). Furthermore, no apparent differences in the Mean *C*_i_/*C*_a_ were detected among the different functional types (Table 3).

### δ^13^C of nighttime leaf-respired CO_2_

We observed a significant nocturnal shift of the δ^13^C of leaf-respired CO_2_ (δ^13^C_R_) in 20 of the 22 species studied, the exceptions being *Chenopodium glaucum* and *Saussurea amara* (Table 4). The leaf-respired CO_2_ at 8 pm was enriched in ^13^C compared with that at 4 am. However, the C_3_ and C_4_ plants showed no statistical significant difference in the amplitude of the nighttime variation in δ^13^C_R_ (Table 3). Moreover, no significant differences in the nocturnal shift in δ^13^C_R_ were detected among the plant functional types (Table 3).

**Table 4:** Carbon isotope composition. The C isotope composition (‰) of leaf-respired CO_2_ (δ^13^C_R_), leaf soluble carbohydrates (δ^13^C_sugar_), leaf starch (δ^13^C_starch_) and leaf lipids (δ^13^C_lipid_) at 8 pm and 4 am, respectively. The carbon isotope composition of leaf bulk tissue (δ^13^C_**biomass**_) was measured once at 8 pm. The amplitude of the nighttime variation in δ^13^C_R_ (Variation in δ^13^C_R_) was estimated as δ^13^C_R-8pm_—δ^13^C_R-4am_. Differences in the δ^13^C_R_ between the samples collected at 8 pm and 4 am were assessed by One-way analysis of variance (ANOVA) and the *P* values are presented.

Species	δ^13^C_R-8pm_ (‰)	δ^13^C_R-4am_ (‰)	Variation in δ^13^C_R_	*P*	δ^13^C_biomass_ (‰)	δ^13^C_sugar-8pm_ (‰)	δ^13^C_sugar-4am_ (‰)	δ^13^C_starch-8pm_ (‰)	δ^13^C_starch-4am_ (‰)	δ^13^C_ipid-8pm_ (‰)	δ^13^C_ipid-4am_ (‰)
*L*. *chinensis*	-22.7±0.17	-24.5±0.16	1.8±0.15	<0.01	-26.6±0.21	-27.4±0.55	-27.9±0.23	-24.7±0.17	-25.3±0.38	-29.9±0.36	-29.3±0.47
*P*. *australis*	-21.7±0.09	-23.5±0.10	1.8±0.12	<0.01	-25.8±0.13	-29.0±0.17	-29.5±0.10	-22.9±0.32	-24.2±0.10	-30.2±0.31	-29.5±0.32
*L*. *bicolor*	-24.9±0.13	-26.6±0.16	1.7±0.26	<0.01	-28.5±0.09	-30.1±0.20	-30.9±0.30	-29.6±0.06	-30.0±0.10	-30.0±0.22	-30.5±0.25
*M*. *asiatica*	-21.2±0.17	-24.7±0.16	3.5±0.32	<0.01	-26.8±0.16	-25.5±0.37	-25.6±0.30	-24.0±0.08	-24.5±0.14	-31.3±0.12	-30.6±0.15
*P*. *simonii*	-22.9±0.24	-25.6±0.17	2.7±0.26	<0.01	-27.8±0.15	-27.4±0.17	-28.5±0.04	-26.1±0.25	-28.5±0.10	-30.6±0.32	-31.8±0.33
*P*. *salicina*	-22.6±0.15	-25.5±0.22	3.0±0.31	<0.01	-26.9±0.18	-25.9±0.26	-25.6±0.08	-25.9±0.21	-25.6±0.16	-30.5±0.13	-29.9±0.17
*C*. *glaucum*	-26.0±0.32	-26.1±0.28	0.2±0.47	0.72	-28.5±0.18	-32.6±0.36	-33.3±0.26	-26.6±0.39	-25.4±0.36	-30.9±0.25	-30.3±0.15
*G*. *max*	-22.6±0.16	-24.7±0.10	2.2±0.25	<0.01	-27.8±0.07	-28.6±0.35	-28.5±0.35	-26.8±0.12	-27.7±0.10	-32.4±0.24	-32.2±0.25
*H*. *annuus*	-26.3±0.31	-28.1±0.26	1.9±0.73	<0.01	-28.2±0.36	-27.7±0.26	-26.8±0.18	-27.0±0.25	-27.6±0.11	-32.3±0.42	-32.1±0.16
*S*. *amara*	-25.5±0.16	-25.0±0.30	0.5±0.32	0.17	-28.4±0.12	-29.1±0.05	-29.4±0.06	-27.0±0.12	-26.8±0.23	-31.4±0.09	-31.8±0.23
*V*. *radiata*	-19.9±0.19	-23.4±0.07	3.5±0.24	<0.01	-26.5±0.09	-27.5±0.04	-27.6±0.15	-26.4±0.07	-26.7±0.11	-33.0±0.32	-30.6±1.06
*V*. *unguiculata*	-23.5±0.32	-27.0±0.41	3.5±0.41	<0.01	-29.3±0.22	-29.3±0.60	-28.1±0.23	-27.4±0.18	-28.8±0.21	-31.1±0.16	-30.8±0.24
*X*. *sibiricum*	-23.7±0.22	-24.6±0.26	1.0±0.41	0.02	-26.2±0.27	-25.5±0.14	-25.3±0.29	-24.9±0.29	-24.4±0.26	-29.5±0.20	-29.1±0.20
*C*. *virgata*	-8.3±0.17	-10.2±0.10	2.0±0.16	<0.01	-12.8±0.26	-15.5±0.24	-15.6±0.32	-11.8±0.07	-12.1±0.38	-21.7±0.28	-21.9±0.36
*E*. *crusgalli*	-9.6±0.12	-11.3±0.08	1.8±0.18	<0.01	-13.2±0.35	-15.7±0.28	-14.1±0.36	-11.6±0.41	-11.2±0.13	-20.1±0.34	-17.9±0.70
*H*. *altissima*	-9.4±0.10	-11.4±0.14	2.0±0.21	<0.01	-12.8±0.31	-15.3±0.27	-15.0±0.21	-11.7±0.16	-12.2±0.13	-21.3±0.18	-21.3±0.28
*P*. *miliaceum*	-8.9±0.33	-13.1±0.14	4.1±0.33	<0.01	-13.3±0.13	-16.0±0.46	-18.1±0.58	-11.4±0.12	-11.9±0.13	-21.3±0.16	-21.1±0.61
*S*. *italica*	-8.6±0.19	-11.9±0.30	3.2±0.39	<0.01	-12.1±0.14	-17.7±0.16	-16.9±0.46	-11.2±0.21	-11.1±0.13	-22.2±0.51	-20.8±0.39
*S*. *viridis*	-7.3±0.14	-9.5±0.17	2.2±0.27	<0.01	-12.2±0.07	-13.5±0.19	-19.4±0.24	-11.4±0.04	-11.8±0.29	-21.1±0.52	-20.5±0.64
*S*. *bicolor*	-9.5±0.13	-12.2±0.20	2.7±0.18	<0.01	-12.3±0.19	-13.2±0.20	-12.9±0.32	-10.8±0.19	-11.5±0.59	-22.0±0.24	-21.2±0.40
*Z*. *mays*	-5.3±0.22	-11.1±0.28	5.8±0.24	<0.01	-12.4±0.19	-14.9±0.71	-13.6±0.28	-10.3±0.09	-11.0±0.44	-22.0±0.68	-21.9±0.35
*A*. *retroflexus*	-7.6±0.26	-12.3±0.31	4.7±0.36	<0.01	-12.2±0.13	-16.6±0.74	-16.5±0.58	-10.8±0.45	-12.4±0.39	-21.0±0.50	-20.9±0.45

Data are reported as the mean ±1 SE (n = 5)

### Correlations between the nocturnal shift in δ^13^C_R_ and cumulative carbon assimilation

**Fig 2:**
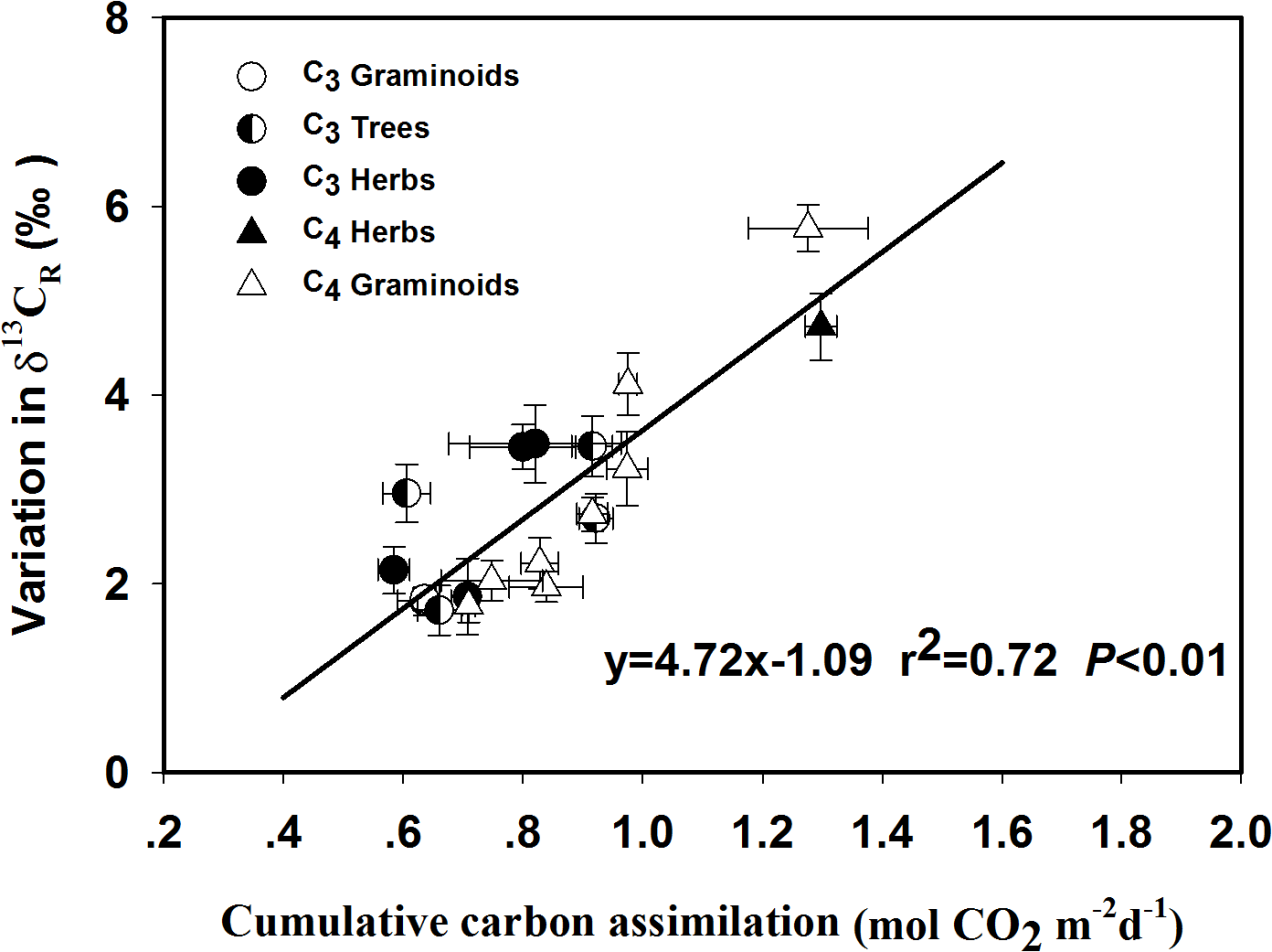
Dependence of the amplitude of the variations in δ^13^C of leaf-respired CO_2_ (δ^13^C_R_, ‰) on the amount of cumulative carbon assimilation (mol CO_2_ m^-2^ d^-1^). The data contains 19 of the 22 studied species, the exceptions being *Chenopodium glaucum*, *Saussurea amara* and *Xanthium sibiricum*. The *r* ^2^ and *P* values are provided. The data are reported as mean ±1 standard error (n = 5).

We calculated the daytime cumulative carbon assimilation, using gas-exchange data to explore the potential effects of the pool size of the daytime cumulative photosynthates on the magnitude of the nighttime variation in δ^13^C_R_. A strong positive correlation was found between the amplitude of the nighttime variation in δ^13^C_R_ and the cumulative carbon assimilation (Fig 2).

### Respiratory apparent ^13^C/^12^C fractionation

**Fig 3:**
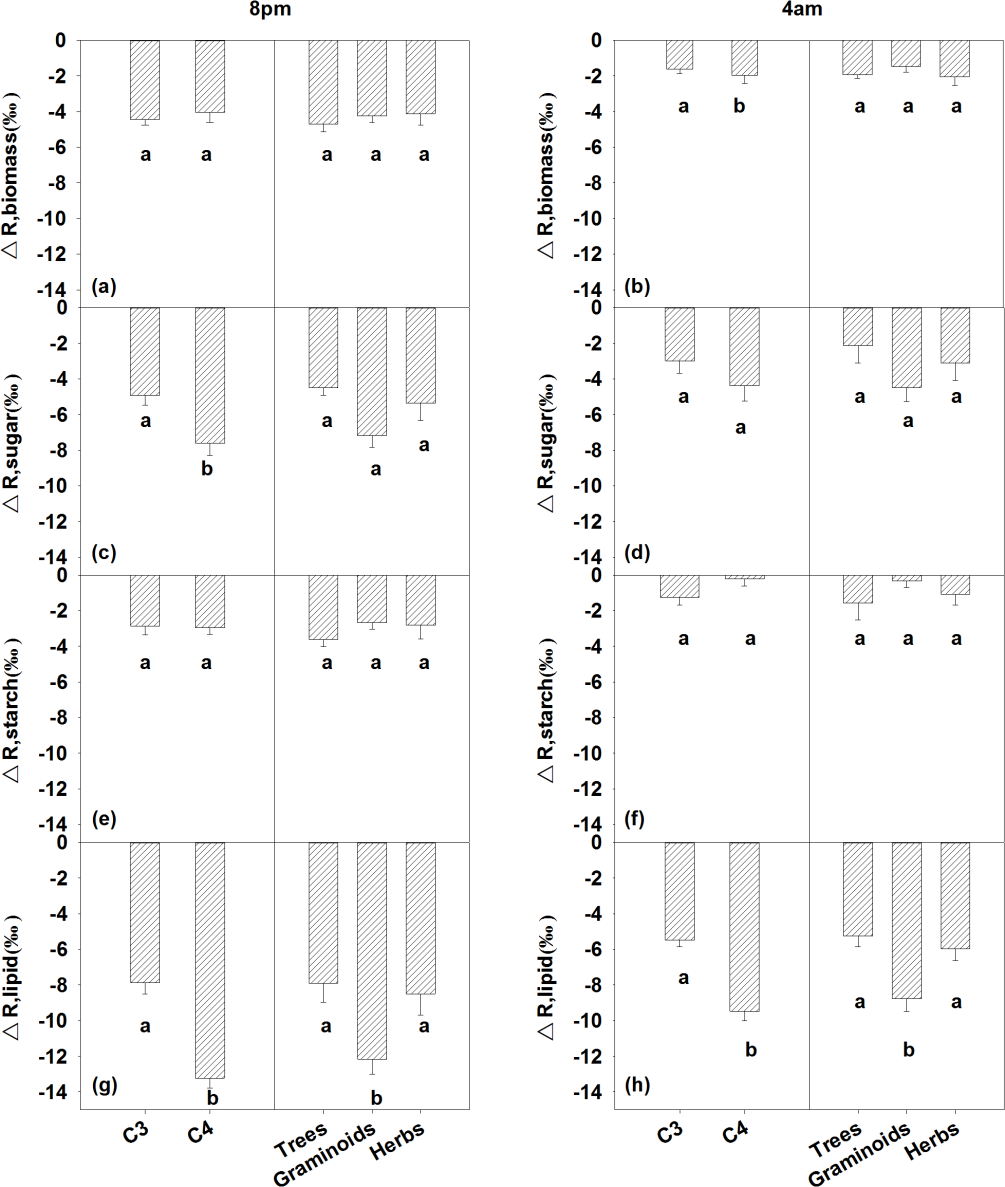
Plant functional type differences in respiratory apparent fractionation comparative to leaf bulk materials (Δ_R, biomass_), soluble carbohydrates (Δ_R, sugar_), starch (Δ_R, starch_) and lipids (Δ_R, lipid_) at 8 pm and 4 am, respectively. Data are reported as mean ± 1 standard error (n = 5). The different letters within each panel indicate the significant differences (*P* <0.05) among the plant functional types.

We calculated respiratory apparent ^13^C/^12^C fractionation (Δ_*R*_) relative to the δ^13^C values of the bulk leaf material, soluble carbohydrates (Δ_R, sugar_), starch (Δ_R, starch_), and lipids (Δ_R, lipid_) at 8 pm and 4 am, respectively (Table 5). Differences in Δ_R, biomass_ between the C_3_ and the C_4_ species were detected at 4 am, but not at 8 pm (Table 3; Fig 3A and 3B). There were no nighttime Δ_R, biomass_ differences among the trees, graminoids and herbs at either 8 pm or 4 am (Table 3; Fig 3A and 3B). Significant differences were found in the Δ_R, sugar_ between the C_3_ and the C_4_ species at 8 pm (Table 3). Neither photosynthetic pathway nor plant functional type differences in Δ_R, starch_ were detected (Table 3). The C_3_ and C_4_ plants differed significantly in the Δ_R, lipid_ at both 8 pm and 4 am (Table 3; Fig 3G and 3H). We also detected plant functional type differences in the Δ_R, lipid_ (Table 3), with the Δ_R, lipid_ values in the graminoids being more negative than they were in the trees or herbs (Fig 3G and 3H).

**Table 5:** Respiratory apparent fractionation. Respiratory apparent ^13^C/^12^C fractionation (‰) of the nighttime leaf-respired CO_2_, comparative to biomass (Δ_R, biomass_), soluble carbohydrates (Δ_R, sugar_), starch (Δ_R, starch_) and lipids (Δ_R, lipid_) at 8 pm and 4 am, respectively.

Species	Δ_R, biomass-8pm_ (‰)	Δ_R, biomass-4am_ (‰)	Δ_R, sugar-8pm_ (‰)	Δ_R,sugar-4am_ (‰)	Δ_R, starch-8pm_ (‰)	Δ_R,starch-4am_ (‰)	Δ_R, lipid-8pm_ (‰)	Δ_R,lipid-4am_ (‰)
*L*. *chinensis*	-4.00±0.28	-2.12±0.27	-4.80±0.69	-3.52±0.28	-2.07±0.48	-0.77±0.52	-7.36±0.51	-4.93±0.60
*P*. *australis*	-4.23±0.63	-2.35±0.23	-7.49±0.20	-6.10±0.12	-1.31±0.33	-0.68±0.16	-8.72±0.34	-6.19±0.31
*L*. *bicolor*	-3.67±0.31	-1.91±0.26	-5.49±0.21	-4.40±0.43	-4.81±0.15	-3.37±0.21	-5.22±0.27	-4.00±0.34
*M*. *asiatica*	-5.72±0.33	-2.19±0.29	-4.42±0.32	-0.97±0.22	-2.90±0.16	0.19±0.23	-10.35±0.20	-6.13±0.13
*P*. *simonii*	-5.02±0.43	-2.67±0.33	-4.62±0.39	-3.05±0.18	-3.27±0.40	-3.02±0.25	-7.90±0.55	-6.41±0.29
*P*. *salicina*	-4.41±0.41	-1.38±0.33	-3.40±0.40	-0.07±0.20	-3.46±0.34	-0.05±0.27	-8.15±0.27	-4.51±0.12
*C*. *glaucum*	-2.60±0.83	-2.44±0.44	-6.86±0.61	-7.41±0.45	-0.70±0.43	0.70±0.37	-5.11±0.45	-4.33±0.40
*G*. *max*	-5.35±0.57	-3.16±0.33	-6.14±0.34	-3.85±0.36	-4.28±0.11	-3.08±0.15	-10.06±0.24	-7.65±0.30
*H*. *annuus*	-1.99±0.57	-0.08±0.48	-1.49±-0.86	-1.35±0.27	-0.77±0.41	0.55±0.35	-6.21±0.59	-4.06±0.11
*S*. *amara*	-3.03±0.52	-3.56±0.96	-3.71±0.14	-4.50±0.32	-1.54±0.23	-1.86±0.10	-6.05±0.20	-7.03±0.41
*V*. *radiata*	-6.73±1.03	-3.22±0.52	-7.76±0.20	-4.31±0.11	-6.75±0.29	-3.36±0.13	-13.3±0.51	-7.39±1.08
*V*. *unguiculata*	-5.90±0.23	-2.34±0.45	-5.93±0.70	-1.09±0.56	-3.99±0.26	-1.84±0.59	-7.74±0.26	-3.92±0.55
*X*. *sibiricum*	-2.60±0.36	-1.62±0.45	-1.92±0.28	-0.73±0.44	-1.25±0.98	0.24±0.37	-5.98±0.39	-4.65±0.36
*C*. *virgata*	-4.42±0.26	-2.47±0.29	-7.21±0.19	-5.32±0.35	-3.49±0.11	-1.78±0.38	-13.49±0.20	-11.70±0.35
*E*. *crusgalli*	-3.68±0.38	-1.89±0.50	-6.21±0.28	-2.78±0.34	-2.02±0.38	0.12±0.06	-10.6±0.33	-6.68±0.68
*H*. *altissima*	-3.44±0.32	-1.39±0.35	-5.94±0.30	-3.58±0.31	-2.31±0.20	-0.72±0.16	-11.97±0.18	-9.93±0.23
*P*. *miliaceum*	-4.37±0.49	-0.22±0.30	-7.13±0.41	-5.12±0.65	-2.46±0.34	1.19±0.21	-12.48±0.26	-8.20±0.74
*S*. *italica*	-3.49±0.87	-0.24±0.39	-8.92±0.49	-5.09±0.64	-2.59±0.23	0.74±0.34	-13.64±0.38	-9.02±0.45
*S*. *viridis*	-4.91±0.20	-2.68±0.19	-10.46±0.21	-9.99±0.19	-4.14±0.12	-2.30±0.18	-13.91±0.50	-11.09±0.59
*S*. *bicolor*	-2.81±0.55	-0.04±0.67	-4.00±0.25	-0.71±0.34	-1.30±0.29	0.74±0.43	-12.6±0.22	-9.11±0.38
*Z*. *mays*	-7.07±0.61	-1.28±0.38	-9.58±0.75	-2.56±0.40	-4.97±0.14	0.11±0.66	-16.8±0.80	-10.88±0.34
*A*. *retroflexus*	-4.72±0.40	-0.04±0.54	-9.07±0.95	-4.31±0.40	-3.24±0.58	-0.08±0.51	-13.53±0.65	-8.69±0.56

## Discussion

### Variation in δ^13^C of leaf-respired CO_2_

For most of the species studied, leaf-respired CO_2_ collected at 8 pm and 4 am differed significant in its carbon isotope composition, with the leaf-respired CO_2_ at 8 pm was enriched in ^13^C compared with the evolved CO_2_ at 4 am (Table 4). This result is consistent with the findings of previous studies [12, 18, 30]. No significant variation in δ^13^C_R_ were detected in two C_3_ herbs (*Chenopodium glaucum* and *Saussurea amara*), which is in agreement with the finding of Priault et al. (2009). However, we observed a significant variation in δ^13^C_R_ in the other three herbs (Table 4) with the magnitude of the nighttime shift was up to 4.7±0.4‰ in a C_4_ herb *Amaranthus retroflexus*. In contrast with the results of Priault et al. (2009), the three tree species we studied showed significant nocturnal shifts in δ^13^C_R_. Large nocturnal shifts in δ^13^C_R_ have also been reported in other tree species, such as *Prosopis velutina* and *Celtis reticulata* [25]. To assess potential plant functional type differences in the magnitude of variation in δ^13^C_R_, we grouped the studied species with significant variation in δ^13^C_R_ according to their differences in photosynthetic pathway and growth form. However, there were no differences in the magnitude of variation in δ^13^C_R_ between the C_3_ and C_4_ plants, as well as among the woody plants, herbs and graminoids (Table 3).

The amplitude of the nighttime variation in δ^13^C_R_ was strongly correlated with the daytime cumulative carbon assimilation (Fig 2). Similar phenomenon has also been reported previously and was mainly attributed to changes in the contribution of light-enhanced dark respiration (LEDR) associated malate decarboxylation to the total respiratory CO_2_ flux [8, 14]. In the light, malate, fixed by phosphoenolpyruvate carboxylase (PEPc), accumulates because of the inhibition of the key respiratory enzymes, such as the mitochondrial isocitrate dehydrogenase, the succinate dehydrogenase, and the 2-oxoglutarate dehydrogenase [16, 37]. In darkness, the mitochondrial malic enzyme and the mitochondrial malate dehydrogenase catalyzed decarboxylation of the ^13^C-enriched malate pool caused the respired CO_2_ to be ^13^C enriched [38]. In a study by Barbour et al. (2011), they reported the effects of LEDR associated malate decarboxylation on ^13^C-enrichment in leaf-respired CO_2_ can last up to 100 min after sunset. For the present study, ^13^C enrichment in leaf-respired CO_2_ at 8 pm (40–50 min after sunset) may partially be attributed to the decarboxylation of malate.

Large diel variation in photosynthetic discrimination (Δ_P_) has also been hypothesized to affect δ^13^C_R_ by changing the δ^13^C of the primary respiratory substrates [18, 28]. We observed no significant differences in the primary respiratory sources (soluble carbohydrates, starch, and lipids) between 8 pm and 4 am. A previous report [24] has also found no apparent variation in the δ^13^C of the leaf primary respiratory substrates. However, we could not discount the effects of the changes in the use of respiratory substrates having different δ^13^C, because we, along with most other studies, measured the δ^13^C of the entire leaf respiratory substrate pools. It is difficult to identify the pool size and the isotopic signature of the fast- and the slow-turnover pools.

Other mechanisms, such as heterogeneous ^13^C distribution within hexose molecules and nighttime variation in the utilization of respiratory intermediates [14, 21], short term variation in carbohydrate pool size and respiration rate, have also been employed to explain nighttime variation in δ^13^C_R_ [15, 16]. However, with the current data set we are unable to test these hypotheses.

### Respiratory apparent ^13^C/^12^C fractionation

Leaf-respired CO_2_ was enriched in ^13^C compared with the biomass, starch, soluble carbohydrates, and lipids (Table 5). This finding is in agreement with the results of previous studies [16, 19, 27, 28, 39, 40]. ^13^C enrichment in leaf-respired CO_2_, relative to the potential respiratory substrates, could be caused by the incomplete oxidation of hexose molecules, which cause a greater ratio of C-3 and C-4 atoms (^13^C enriched compared with other C atoms) being converted to CO_2_ [30, 37]. However, there were some differences in the magnitude of the respiratory apparent fractionation between the C_3_ and C_4_ species, and among the different plant functional types (Fig 3, Table 3). Differences in Δ_R, biomass_ were detected between C_3_ and C_4_ species at 4 am, but not at 8 pm, which were caused primarily by different magnitude of variation in δ^13^C_R_ between the two photosynthetic types. In a recent review paper, Ghashghaie & Badeck (2014) reported that C_3_ and C_4_ species differed in Δ_R, biomass_. There were no differences in the Δ_R, biomass_ among the trees, the herbs, and the graminoids (Fig 3A), which is in line with the results of Ghashghaie & Badeck (2014). The detected differences in Δ_R, biomass_ between C_3_ and C_4_ species needs to be incorporated into the partitioning of the CO_2_ exchange between the ecosystem and the atmosphere when leaf bulk materials are used as a proxy for δ^13^C_R_ [25].

Δ_R, lipid_ significantly differed between the C_3_ and the C_4_ plants, as well as among the plant functional types (Table 3), which could have resulted primarily from the carbon allocation differences associated with the plant functional group types. Compared with the woody plants or the herbs, the grasses allocated a smaller proportion of acetyl CoA to the synthesis of lipids. This leads to grass lipids being generally more depleted compared with the bulk tissue [26, 41].

Soluble carbohydrates are often found to be ^13^C-enriched compared to bulk leaf material [9, 15, 28]. However, we observed that soluble carbohydrates in 16 of the 22 studied species were depleted in ^13^C relative to the leaf biomass (Table 4). Similar results with up to 4‰ depletion in soluble carbohydrates compared to bulk materials have also been reported previously in various C_3_ and C_4_ species [3535, 36, 42]. This discrepancy may be attributed to both differences in the extraction method and timing of leaf sample collection.

## Conclusions

For both the C_3_ and the C_4_ species (except *Chenopodium glaucum* and *Saussurea amara*), the δ^13^C of leaf-respired CO_2_ (δ^13^C_R_) showed a significant nocturnal shift. The leaf-respired CO_2_ at 8 pm was enriched in ^13^C, relative to what it was at 4 am. The amplitude of the nighttime variation in δ^13^C_R_ was strongly correlated with the daytime cumulative carbon assimilation, which suggests that variation in δ^13^C_R_ were influenced, to some extent, by changes in the contribution of malate decarboxylation to total respiratory CO_2_ flux. There were no differences in the magnitude of the nocturnal shift in δ^13^C_R_ between the C_3_ and C_4_ plants, as well as among the woody plants, herbs and graminoids. The plant functional group differences in respiratory apparent fractionation relative to biomass indicate that caution should be exercised when the δ^13^C of bulk leaf material is used as a proxy for the δ^13^C of leaf-respired CO_2_.
